# Synergistic effects of Al, Ga, and In doping on ZnO nanorod arrays grown via citrate-assisted hydrothermal technique for highly efficient and fast scintillator screens

**DOI:** 10.1186/s11671-025-04227-5

**Published:** 2025-07-11

**Authors:** Murat Kurudirek, Sinem V. Kurudirek, Anna Erickson, Nolan Hertel, Benjamin J. Lawrie, Yauhen Tratsiak, Benjamin Klein, Charles L. Melcher, Christopher J. Summers, Paul J. Sellin

**Affiliations:** 1 Department of Physics, University of Surrey, Guildford, GU2 7XH UK; 2 Nuclear and Radiological Engineering Program, Georgia Institute of Technology, Atlanta, GA 30332 USA; 3 Department of Electricity and Energy, Technical Sciences Vocational College, Ataturk University, 25240 Erzurum, Turkey; 4 Materials Science and Technology Division, Oak Ridge National Laboratory, Oak Ridge, TN 37831 USA; 5 Center for Nanophase Materials Sciences, Oak Ridge National Laboratory, Oak Ridge, TN 37831 USA; 6 Scintillation Materials Research Center, University of Tennessee, Knoxville, TN 37996 USA; 7 Southern Polytechnic College of Engineering and Engineering Technology, Kennesaw State University, Marietta, GA 30060 USA; 8 Department of Nuclear Engineering, University of Tennessee, Knoxville, TN 37996 USA; 9 Department of Materials Science and Engineering, University of Tennessee, Knoxville, TN 37996 USA; 10 School of Materials Science and Engineering, Georgia Institute of Technology, Atlanta, GA 30332 USA

**Keywords:** ZnO nanorods, Scintillation detectors, Ultrafast scintillators, Alpha particles

## Abstract

To be used as efficient alpha particle scintillator in the fields of nuclear security, nuclear nonproliferation and high-energy physics, scintillator screens must have high light output and fast decay properties. While there has been a great deal of progress in scintillation efficiency, achieving fast decay time properties are still a challenge. In this work, the near band edge (NBE) UV luminescence and alpha particle induced scintillation properties of vertically aligned densely packed ZnO nanorods (NRs) doped with Al, Ga, and In have been thoroughly investigated. The high crystalline hexagonal wurtzite structure with a strong orientation through the *c*-axis plane (002) and aspect ratios in the range 13–22 have been observed for all ZnO NRs. Electron paramagnetic resonance (EPR) analysis exhibited paramagnetic signals at *g *≈ 1.96 for all ZnO NRs. A cost effective green hydrothermal synthesis technique was employed to grow well-aligned NRs. Using citrate as an additive acting as a strong reducing agent in the solution during the crystal growth, defects on the surface are significantly suppressed, thereby enhancing the NBE UV emission. Significantly higher NBE UV emission was observed from the top surface of ZnO NRs in cathodoluminescence (CL) microscopy. Results show that citrate assisted donor doping of ZnO NRs not only reduces the defect emission and NBE self-absorption, but also induces fast decay time (~ 600–700 ps), which makes ZnO NRs a good candidate for fast alpha particle scintillator screens used in associated particle imaging for time and direction tagging of individual neutrons generated in D–T and D–D neutron generators.

**Supplementary Information:**

The online version contains supplementary material available at 10.1186/s11671-025-04227-5.

## Introduction

Among different types of radiation detector materials, scintillators are widely used as UV, X-ray imaging and alpha particle detectors. A scintillator material absorbs high energy photons and charged particles, and subsequently emits scintillation photons in the UV or visible spectrum. One possible application of alpha particle scintillators is the use of an alpha particle scintillation screen with high spatial resolution, fast temporal resolution, and a high signal-to-noise ratio which could be utilized as a “fingerprint” technology in the field of explosive detection i.e. the deuterium–tritium (D–T) reaction associated particle neutron generator (APNG) [1]. While excellent progress has been made in scintillation efficiency for traditional scintillators such as CsI(Tl), the fast time performance is still challenging [2]. ZnO, on the other hand, is one of several direct band gap semiconductors with a wide band gap (3.3 eV) at room temperature [3], a large exciton binding energy (60 meV), and a high light output with a sub-nanosecond decay time [1, 2, 4–11]. Therefore, it is a good candidate for an alpha particle scintillator. Doping a wide bandgap semiconductor with various n-type dopants can improve its optical and structural properties by generating a metastable population of electrons near the bottom of the conduction band [8]. When doped with donors such as Al, Ga, and In, ZnO displays a sub-nanosecond decay time making it a potential super-fast scintillator with potential use in medical physics, homeland security, nuclear nonproliferation, and nuclear/high energy physics. Therefore, it is important to understand the effects of the dopants on the structural, optical, and scintillation properties of ZnO nanorods (NRs) prior to employing these materials in practical applications [12].

ZnO photoluminescence (PL) spectra typically includes near band edge (NBE) UV excitonic emission and visible emission originating from defects in the crystal structure. Two typical emission has been observed in 1D ZnO nanostructures [13, 14] and the detailed NBE PL studies on 1D ZnO nanostructures revealed a donor-bound exciton emission, and emissions related to the donor–acceptor pair (DAP) transition and the longitudinal optical phonon replicas of the DAP transition [15, 16]. NBE UV emission intensity (with a blue shift in peak wavelength when doped with Ga and In) has been shown to decrease when ZnO was doped with 2% Ga and In, while an increase in visible emission intensity has been observed for In doped ZnO NRs and a decrease in visible emission has been observed for Ga doped ZnO NRs [17]. In another study from the same research group, similar variations (except for an anomalous red shift in NBE UV emission for doped ZnO NRs) in NBE UV and visible PL were reported for ZnO doped with Ga and In [18]. This change was attributed to the nanorod size, band gap renormalization and stacking faults induced by dopants. A red shift observed in NBE UV emission of In doped ZnO NRs along with an increase in visible emission was attributed to the Mott critical density depending on the carrier concentration in the structure [19, 20]. However, the UV NBE emission was found to be more or less the same for ZnO, ZnO:Ga, and ZnO:In samples and the visible emission of undoped ZnO NRs was weak when compared to doped ZnO [12]. In another study, ZnO nanostructures grown by vapor phase transport process exhibited higher visible emission when doped with Ga and In [21]. Moroever, violet emission has been observed in different ZnO nanostructures, which might be due to zinc vacancies, and are considered stable defects [22, 23]. While an agreement on the origin of the visible emission in ZnO has yet to be reached, there are possible causes for this phenomenon such as oxygen vacancy (V_O_), zinc vacancy (V_Zn_), oxygen interstitial (O_i_), zinc interstitial (Zn_i_), oxygen anti-site (O_Zn_), and zinc anti-site (Zn_O_) defects [24]. In addition to PL investigations, electron paramagnetic resonance (EPR) spectroscopy has been used to monitor different paramagnetic defect states [25–31]. Two intrinsic paramagnetic defects in ZnO nanostructures have been reported with the corresponding *g*-factor values as *g* (I) ≈ 2.00 and *g* (II) ≈ 1.96 [25, 32]. These EPR signals were referred to oxygen vacancies capture of one electron whereas the latter one also was ascribed to shallow donors [25, 26, 30]. Al, Ga or In are considered substitutional impurities forming shallow donors in single crystals of ZnO by replacement of Zn atoms [29, 33, 34].

ZnO nanostructures and composites were found to have very promising alpha particle induced scintillation properties [1, 2, 7, 35–38]. Besides this, the ZnO nanostructures were also studied for their X-ray and UV detection properties as well [39–41]. In order to suppress the defect emission due to oxygen vacancies in ZnO NRs, high temperature hydrogen annealing has been used [1]. However, it is important to maintain the crystal structure of ZnO, which can be challenging in high temperature annealing. One other option could be using different additives in solution-based growth techniques to suppress defect related emission in the structure. Addition of sodium citrate in the solution could help suppress deep level emission in ZnO nanostructures. However, citrate assisted growth of ZnO nanostructures has resulted in loss of the nanorod structure and poor alignment through the *c*-axis [42, 43]. Therefore, to obtain well-aligned and high efficiency NBE UV ZnO NR emitters, more studies on additive optimization and relatively low temperature annealing are required. In the present work, we introduce a citrate assisted simple, cost effective, less-toxic hydrothermal growth of vertically well-aligned Ga, In, and Al doped tapered ZnO NRs with fast exciton dynamics suitable for alpha particle scintillators using a one cycle growth with negligible defect related visible emission before and after annealing. The effect of doping on crystal quality, NBE UV emission and scintillation properties is also discussed.

## Experimental section

Tapered ZnO NRs were synthesized in two steps. First step is the deposition of a ~ 100 nm thick ZnO seed layer on silica glass substrate (MSE Supplies LLC) by sputtering. Substrates were 0.5 mm thick and had ~ 2.5 cm × 2.5 cm dimensions. An RF sputtering technique was used with Ar ions in a chamber to sputter ZnO clusters and deposit them on the substrate forming a continuous film. Silica glass substrates coated with ZnO seed layer were placed in a solution prepared with zinc nitrate (Zn(NO_3_)_2_.6H_2_O, Sigma Aldrich, 98%), HMTA (C_6_H_12_N_4_, Sigma Aldrich, ≥ 99%), ammonium hydroxide (NH_4_OH, Sigma Aldrich), and trisodium citrate (Na_3_C_6_H_5_O_7_, Sigma Aldrich, ≥ 99%), along with gallium (III) nitrate hydrate (Ga(NO_3_)_3_·xH_2_O), indium (III) nitrate hydrate (In(NO_3_)_3_·xH_2_O), or aluminum (III) nitrate nonahydrate (Al(NO_3_)_3_·9H_2_O) as dopants. After addition of dopant compounds in to the base solution, the solution stirred for 20 min using magnetic stirring to obtain a homogeneous solution. Nitrate based compounds, which are highly water soluble, were used in growth solution. The zinc nitrate to HMTA ratio was 2:1. Doping concentrations of Al, Ga, and In ratios were kept 1% (mol) relative to Zn. The ZnO NRs were grown for 25 h by maintaining the oven temperature at 95 °C. After the growth, the substrate was rinsed with DI water and was dried at room temperature. Samples were then annealed in a 10%H_2_ atmosphere for 1 h at 350 °C. The kinetics of the growth of ZnO NRs is given in detail elsewhere [11]. Polar planes (001) of ZnO consisting of either Zn or O atoms stimulate the *c*-axis growth since they have higher surface energies than the other non-polar planes, (110) and (100). The NH_4_^+^ ions supplied from HMTA adsorb on the lateral surfaces and suppress lateral growth since the adsorption of Zn^2+^ ions on the lateral surfaces is inhibited [18].

For characterization measurements, X-ray diffraction (XRD), scanning electron microscopy (SEM), photoluminescence (PL, Renishaw spectrometer attached with a He-Cd 325 nm laser), X-ray photoelectron spectroscopy (XPS), cathodoluminescence (CL), and scintillation spectroscopy were used. SEM (HITACHI SU8230) and XRD (Panalytical XPert PRO) instruments were used to characterize the structural properties of the ZnO NRs, and the optical measurements were performed using a He-Cd 325 nm UV laser and a PL spectrometer. The structure of chemical bonding was analyzed by X-ray photoelectron spectroscopy (Thermo NEXSA G2 XPS) utilizing Al K alpha X-rays. The C 1 s spectrum of carbon (BE ~ 285 eV) was used as a reference to correct the charge offset based shift of binding energies. The absorbance of the samples was measured using a Cary 5000 UV/Vis/NIR spectrophotometer with tungsten halogen and deuterium arc light source. Room temperature EPR measurements were carried out using the ELEXSYS-II E500 X-band (≈ 9.8 GHz) CW (continuous wave) EPR spectrometer from Bruker (Mod Freq. @ 100 kHz, microwave power @ 0.25 mW, modulation amplitude @ 1.0 G). Each sample in the resonator was critically coupled to the emitted microwaves to ensure maximum signal detection during a resonance absorption. Samples were annealed in a tube furnace (Thermolyne 21,100 Tube Furnace). Alpha particle Pulse Height Distribution (PHD) measurements were conducted using alpha particles of ~ 5.5 MeV from an ^241^Am radioactive source and a PMT (Hamamatsu).

A Horiba Jobin Yvon Fluorolog 3 spectrofluorometer with a Xe lamp and dual scanning monochromators was used for steady-state PL emission and excitation measurements. In order to block the second order excitation wavelength a 320 nm optical filter was used. Low temperature PL measurements were conducted using an optical O-ring sample holder on which the sample sit between two silver foil pieces having a window from one side. The cold finger of a DE202AE cryostat was used as an attachment to the sample holder. A Lakeshore 331 Temperature Controller was used to adjust temperature manually. A 380 nm optical filter was used for blocking the second order excitation wavelength.

A FEI Quattro environmental SEM with an accelerating voltage of 5 kV and a beam current of 14 pA was used in CL measurements under a 250 mTorr water vapor background to suppress charging. These beam conditions did not cause any sample degradation after CL measurements. All the SEM and CL measurements of the samples were stable. The samples exhibited no visible beam-induced degradation, and the CL response was consistent across different areas sampled on each chip. In order to collect the light produced by the samples a Delmic Sparc cathodoluminescence module was used including a parabolic mirror with a numerical aperture of 0.97 to collimate the light and direct it out of the SEM chamber, where it was focused onto the slit of an Andor Kymera 193 spectrograph and measured on an Andor Newton CCD camera. Time resolved cathodoluminescence was measured using a pickoff mirror that directed the collimated light into a 30-micron core fiber. A 50/50 fiber splitter was used to create a fiber-optic Hanbury Brown-Twiss interferometer, with photons detected on a pair of superconducting nanowire single photon detectors and time tagged using a Hydraharp time tagger as previously described. The excited state dynamics were then extracted from the relaxation of the measured photon bunching [44, 45].

## Results and discussion

**Fig. 1:**
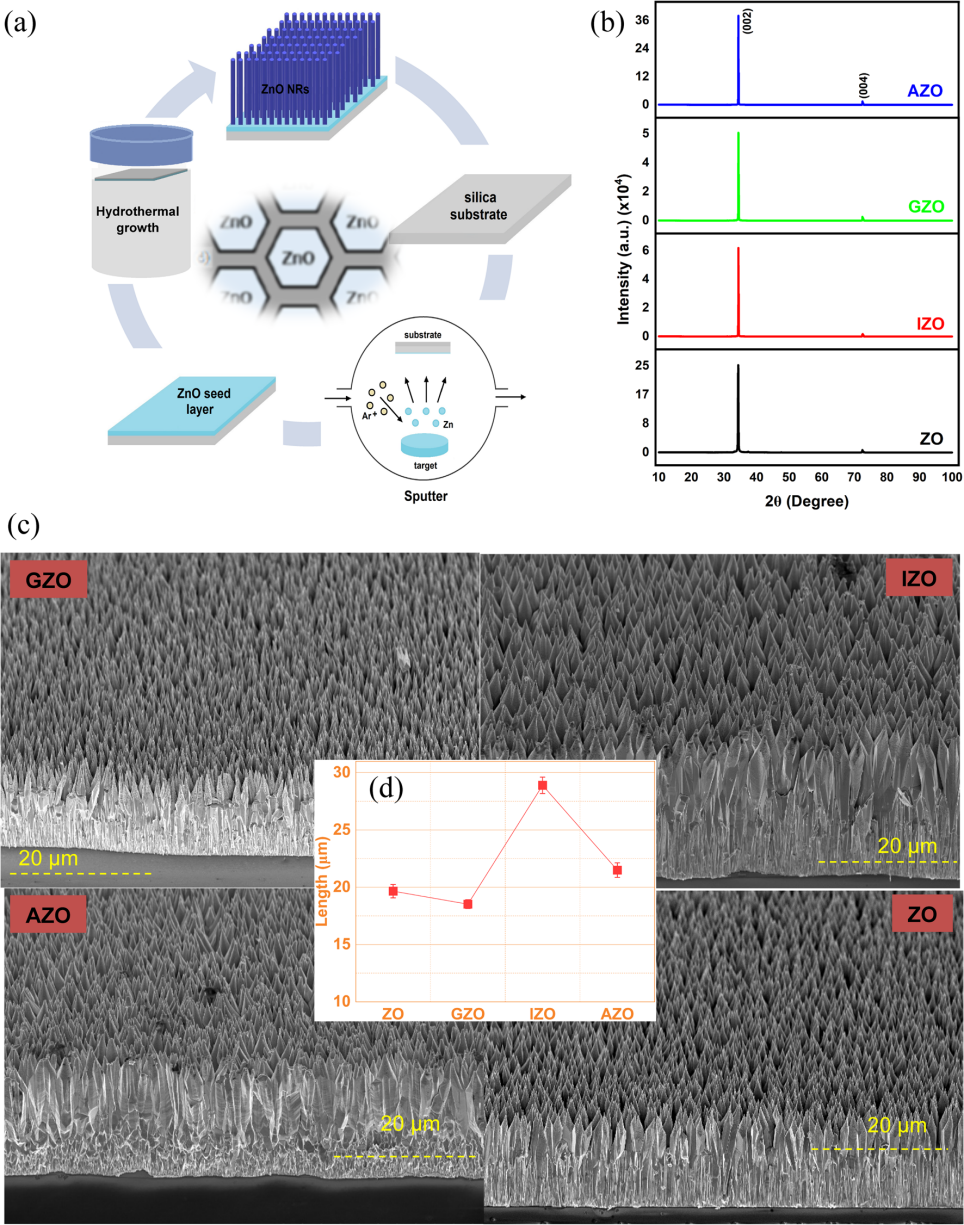
**a** Growth diagram of ZnO NRs using hydrothermal synthesis. **b** XRD patterns of as grown ZnO NRs (ZO) and In, Ga and, Al doped ZnO NRs (IZO, GZO and AZO). **c** Tilted side view SEM images of ZO, GZO, IZO, and AZO. **d** Length of the ZnO NRs

The growth diagram of ZnO NRs using hydrothermal synthesis is depicted in Fig. 1a. The XRD diffraction pattern of the undoped ZnO (ZO) and doped ZnO NRs confirms a wurtzite structure with a strong orientation through the *c*-axis plane (002) along with a minor contribution from other directions i.e. (004) (Fig. 1b). The observed strong and sharp peaks (002) demonstrate the high crystalline quality and formation of ZnO:Al (AZO), ZnO:Ga (GZO), and, ZnO:In (IZO) structures and match well with the standard powder diffraction data (JCPDS, now ICDD, card No. 36–1451) [1, 12, 46–48]. It is important to note that any secondary peaks of Al_2_O_3_, Ga_2_O_3_ or In_2_O_3_ were not observed in the XRD patterns and the hexagonal wurtzite phase of the host matrix is well maintained in the doped ZnO NR samples.

The SEM images clearly show evidence of densely grown vertically well-aligned nanostructured morphology of the grown ZnO NRs (Fig. 1c). A hexagonal cross section tapered ZnO NR stem is clearly seen from surface morphologies. The aspect ratios for the ZnO NRs were found to be 22, 13, 14, and 16 for GZO, IZO, AZO, and ZO, respectively. The tapering tips were excluded in aspect ratio calculations. Highest aspect ratio was observed for GZO NRs while IZO and AZO NRs have the lowest aspect ratio due to the faster lateral growth rate than ZO and GZO NRs. Tapering of the ZnO NRs towards the end of the structure can be better seen in Fig. S1. The length of the NRs ranges between 18 and 28 µm (Fig. 1d). Faster lateral growth has been observed for AZO and IZO NRs while GZO NRs seem to have similar NR structure with ZO NRs indicating that the incorporation of different dopants such as Al, Ga, and In has an effect on the morphology of ZnO NRs to a more or less extent. The difference in atomic radii between undoped and doped ZnO crystals could explain the observed diameter variation in these structures. The atomic radii of Al^3+^ (0.54 Å) and Ga^3+^ (0.62 Å) are smaller than that of Zn^2+^ (0.74 Å), while the atomic radii of In^3+^ (0.81 Å) is greater than that of Zn. Incorporation of Ga in ZnO was reported to cause negligible lattice distortion [46]. Similar increase in lateral growth rate for AZO NRs has been reported elsewhere [47]. Significant increase in lateral growth for the doped ZnO NRs might be due to both the low density of ZnO nuclei due to the inhibited heterogeneous ZnO nucleation and the coordination of dopant ions with OH^−^ ions reducing reaction kinetics between Zn^2+^ and OH^−^ [18]. The highest length observed in IZO NRs might be due to the interstitial reactions between Zn and In atoms which have different atomic radii [12].

**Fig. 2:**
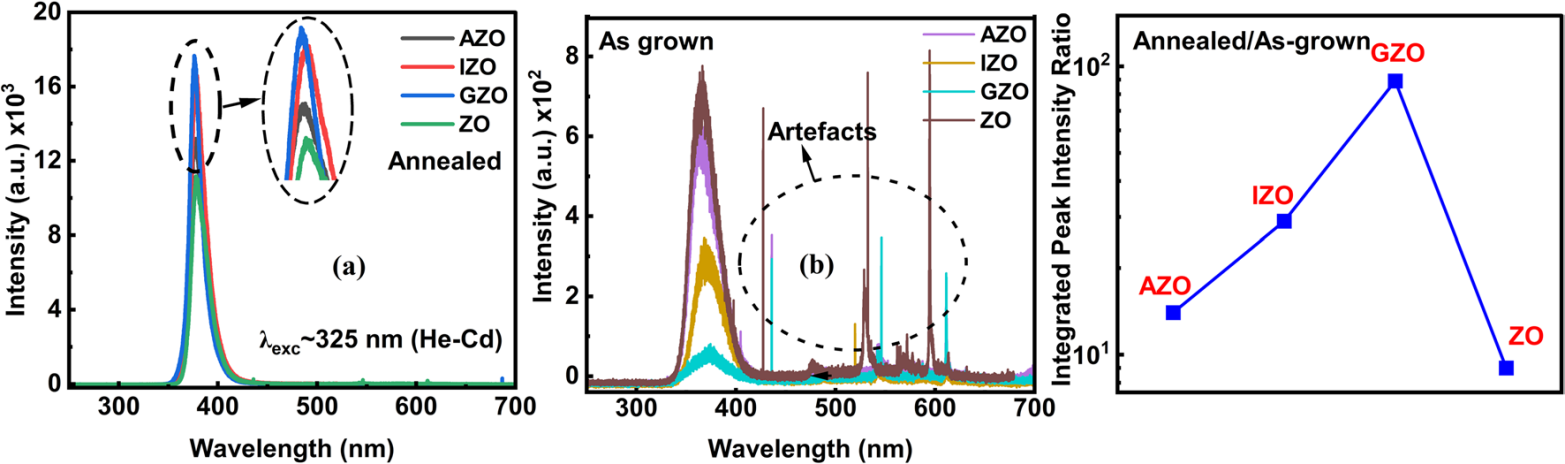
PL spectra of ZnO NRs using 325 nm excitation wavelength of a He–Cd laser: **a** after annealing **b** before annealing **c** Integrated PL peak intensity ratio of annealed to as grown ZnO NRs

The room temperature PL measurements were performed using a 325 nm He–Cd laser to investigate the NBE UV emission from ZnO NRs (Fig. 2). The laser beam intensity was optimized using different density filters and measurements were repeated in different spots on the sample indicating no significant change in PL intensity (Figs. S3, S4). After annealing in a forming gas atmosphere, all ZnO NRs showed significantly enhanced UV emission (~ 377 nm) when compared to the as-grown samples (Fig. 2a, b). By annealing samples in a hydrogen atmosphere, the O vacancies, which cause surface defects and reduce the UV emission, are substituted by hydrogen, which create shallow donor levels close to the conduction band [1, 49–51]. Therefore, the substitutional H in ZnO structure creates hydrogen-donor-bound exciton levels that enhance the NBE UV emission [52]. The NBE UV PL peak intensity ratios of annealed to as-grown ZnO NRs indicate that the UV emission is significantly enhanced in doped ZnO NRs (Fig. 2c). The enhancement factor of integrated UV peak intensities (annealed/as-grown) varies between ~ 9 (ZO) and ~ 89 (GZO) and was found to be always higher in doped ZnO NRs than in as-grown ZnO NRs.

GZO NRs have shown the highest NBE UV emission amongst other ZnO NRs which might be attributed to the negligible adverse effect of Ga on ZnO NR lattice structure and crystallinity or reduced oxygen vacancies leading to native defect quenching and increased non-radiative defect centers [46, 53]. Even before annealing, the surface-defect related emission in the visible region seems negligibly low. This is due to a strong reducing agent, sodium citrate, used in the NR growth solution [11]. As is discussed below, the NBE UV emission mainly originated from the top polar surface (0001) of ZnO NRs. In contrast, the visible emission due to the surface defects is seen from the lateral non-polar surface (1010) of the ZnO NRs [39, 54]. The negative citrate ions tend to adsorb on the positive polar surface (0001) of the ZnO NRs and binds Zn^2+^ ions as a chelating agent [55, 56]. This would lead ZnO_2_^2−^ ions to selectively adsorb on the lateral surfaces of the ZnO NRs which could increase the lateral growth rate [57, 58]. Also, an increase in the density of neutral Zn atoms could increase the density of Zn interstitials and therefore reduce the density of O vacancies, thus suppressing the visible emission in ZnO NRs [59]. The UV–visible absorption spectra of ZnO NRs are given in Fig. S2d. ZO, GZO, IZO and AZO NRs had major absorption peaks in the UV region. Absorption peaks of doped ZnO NRs (dominated by CB-VB transition) were all red-shifted when compared with the as grown ZnO NRs. Using the Tauc plot of (*α*hv)^2^ as a function of the photon energy, the optical band gap of the ZnO NRs was estimated (Fig. S2d inset) [60]. The band gaps were calculated as 3.19, 3.24, 3.22, and 3.20 for IZO, GZO, AZO, and ZO, respectively.

**Fig. 3:**
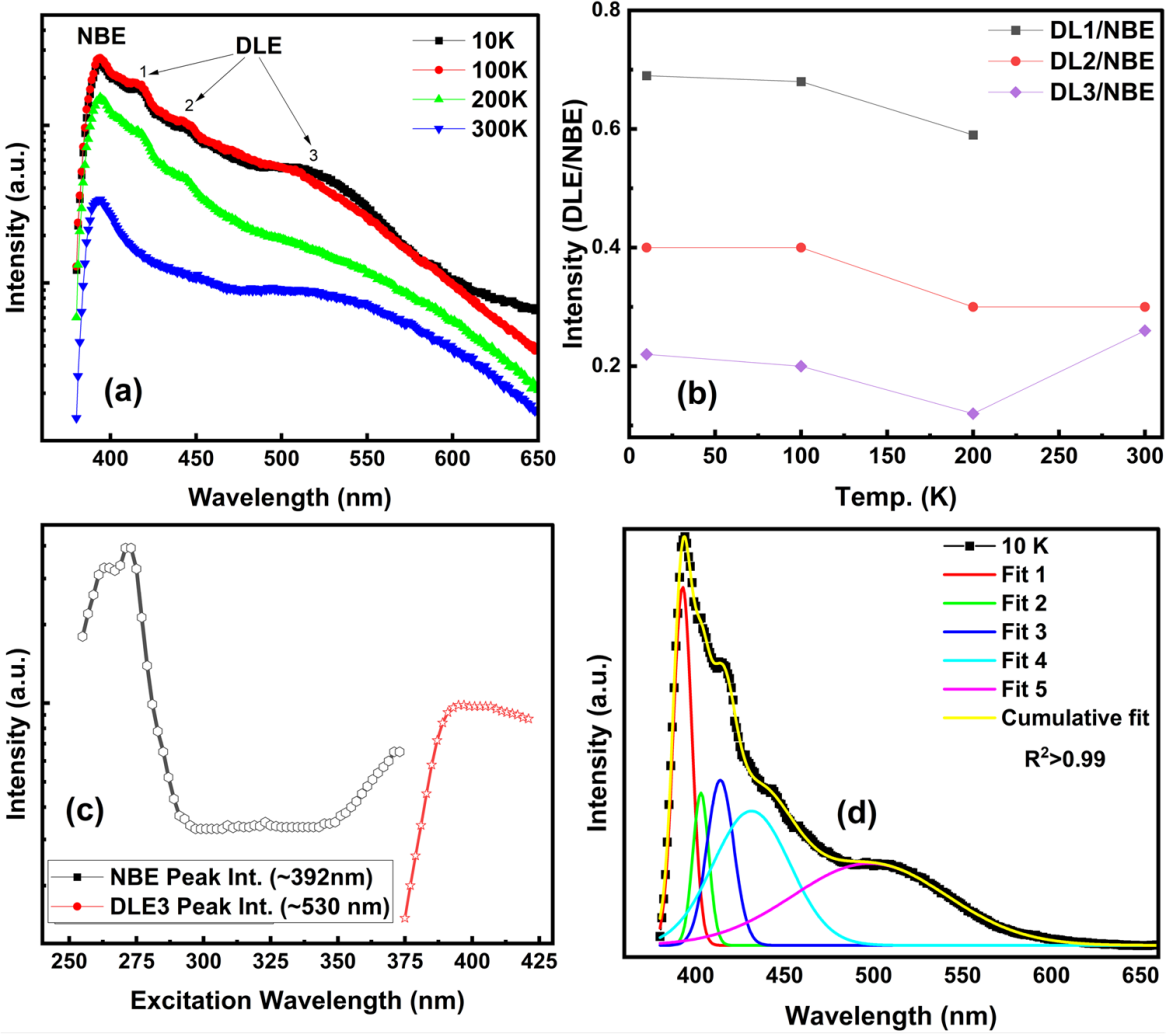
Steady state PL spectra of as grown ZnO (ZO) NRs: **a** Temperature-dependent PL spectra at 325 nm excitation wavelength including NBE emission and DLE; **b** DLE to NBE peak ratio of ZO NRs depending on the temperature; **c** Variation of room temperature NBE and DLE peak intensities with excitation wavelength; and **d** Deconvolution of the PL spectra at 10 K

To further analyze the temperature dependent excitonic NBE emission and defect related deep level emission (DLE) characteristics of the ZnO NRs, steady-state PL measurements were conducted for as-grown ZnO NRs using a Xe lamp as an excitation source. Emission spectra consist of a strong NBE emission corresponding to excitons bound to neutral and ionized donors and weak DLEs corresponding to defect related emissions (Fig. 3a) [61, 62]. A strong UV emission was observed at ~ 392 nm regardless of temperature while weak violet visible emissions were observed up to 200 K between ~ 400–450 nm. A decrease in violet emission with increasing temperature has been reported before and the Zn interstitials (Zn_i_) present in the structure might cause these weak emissions at lower temperatures by forming shallow traps for excited states [63, 64]. The disappearance of the violet peak above 200 K might be due to an enhanced transition from the Zn_i_ level to the oxygen vacancy (V_O_) level which would enhance green emission or thermal quenching of the emission center [64]. A weak green emission band around ~ 530 nm was also observed, possibly due to the O vacancy related defect emission [39]. The UV emission is quenched with increasing temperature due to the thermal ionization of bound excitons [65, 66]. The intensity ratios of DLE to NBE emission with varying temperature are shown in Fig. 3b. It has been seen that violet emission dominates at low temperatures compared to the green emission and tends to decrease with increasing temperature as explained above. For better understanding of the temperature dependent emission mechanism, further research on the wavelength resolved thermally stimulated luminescence (TSL) measurements is required [67]. On the other hand, green emission was found to be highest at room temperature as expected. Room temperature steady state PL data for ZnO NRs are also provided in Fig. S5.

Figure 3c shows the room temperature PL excitation spectrum of NBE (~ 392 nm) and visible emission (DLE3 ~ 530 nm) as a function of excitation wavelength (*λ*_exc)_. The intensity of NBE UV starts increasing up to around *λ*_exc_ ~ 270 nm and then decreases until *λ*_exc_ ~ 295 nm. There the NBE PL excitation efficiency plateaus from *λ*_exc_ ~ 295–350 nm. Thereafter it starts increasing again until *λ*_exc_ ~ 373 nm, i.e. the optical absorption edge for ZnO NRs. The visible emission intensity starts increasing in the range *λ*_exc_ ~ 375–393 nm and then starts slightly decreasing. Spectral decomposition of NBE and visible emission spectra is shown in Fig. 3d. Deconvolution of the low temperature spectra resulted to a dominant NBE UV peak (~ 392 nm) along with weak violet emissions centered at ~ 402, ~ 413, and ~ 431 nm and a green emission at ~ 498 nm. Weak violet emission could be attributed to the Zn vacancy and interstitial Zn related defects in the structure [68, 69]. The energy levels of native defects in ZnO NRs based on Kröger Vink notation has been depicted in Fig. S6. The donor related defects such as ${Zn}_{i}^{\bullet \bullet } and {Zn}_{i}^{\bullet }$ lead to violet emission which could originate from exciton recombination between electrons located at ${Zn}_{i}^{\bullet \bullet } and {Zn}_{i}^{\bullet }$ levels and valence band [63, 69, 70]. ${V}_{Zn}^{^{\prime\prime} }$ constitutes a shallow acceptor defect located ~ 0.3 eV above the VB maximum, which can be easily formed in n-type samples and is attributed to the recombination of an electron with a deep level trapped hole in the ${V}_{Zn}^{^{\prime\prime} }$ center [27, 68]. Finally, the green luminescence which might be related to the oxygen vacancy defect could originate from the electron transition from singly ionized ${V}_{O}^{\bullet }$ state to the valence band [49, 71]. Variation of NBE UV emission with *λ*_exc_ is shown in detail in Fig. S7. The UV intensity grows initially as *λ*_exc_ increases until 273 nm (Fig. S7a) and then decreases with a slight blue shift in peak position up to *λ*_exc_ ~ 299 nm (Fig. S7b). Variation of PL emission until *λ*_exc_ ~ 299 nm could be attributed to the contributions of different emission centers. Increasing violet emission results in an apparent increase in the NBE linewidth as the excitation wavelength increases until *λ*_exc_ ~ 273 nm, though this effect reverses as the excitation wavelength continues to increase until the violet emission disappears at around *λ*_exc_ ~ 299 nm. The variation in intensity becomes negligible between *λ*_exc_ ~ 300 and *λ*_exc_ ~ 350 nm (Fig. S7c) and the NBE UV intensity starts increasing for longer wavelengths with no change in peak position (Fig. S7d).

**Fig. 4:**
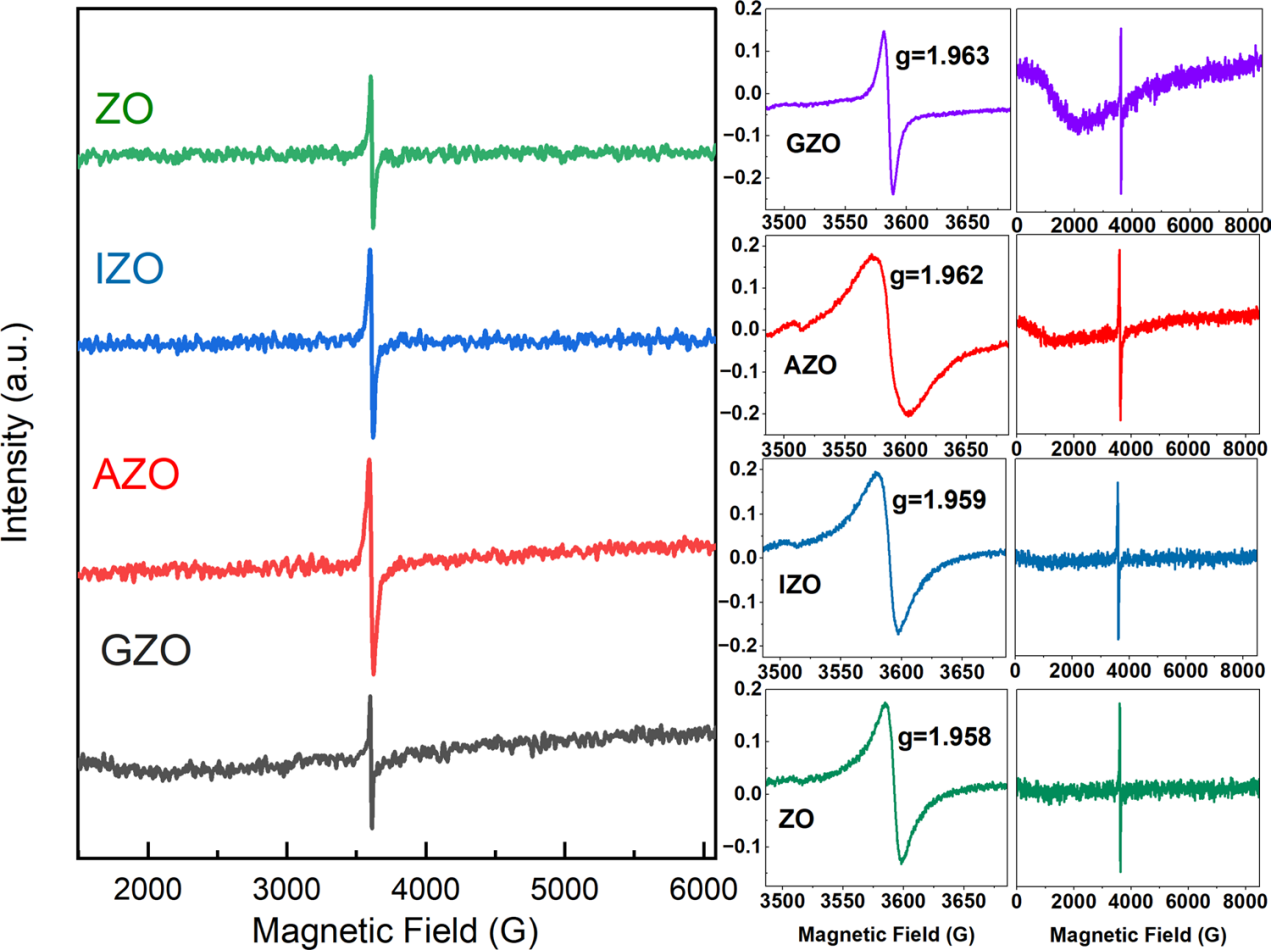
Room temperature EPR spectra of the as grown and doped ZnO NRs. Raw spectra are shown (left) along with the magnified spectra including *g*-factor values (right)

The room temperature EPR measurements were performed for further defects analysis. The EPR results of ZnO NRs are shown in Fig. 4. All ZnO NRs showed a major signal at around *g* = 1.96 with more or less the same intensity. Two signals corresponding to *g* (I) ≈ 2.00 and *g* (II) ≈ 1.96 are well-studied ZnO EPR signals. The latter one is generally referred to different origins such as singly ionized positive oxygen vacancies, [27, 31, 72, 73] zinc interstitials [74], shallow donors [32, 75–77] whereas the EPR signal at *g* ≈ 2.00 is attributed to the Zn vacancy [26], O vacancy [27, 28, 78], and surface defects [25]. EPR studies on doped ZnO have ascribed the EPR signal at *g *≈ 1.96 as shallow donors [26, 30] and the EPR signal at *g* ≈ 2.00 as O vacancy and interstitials as mentioned above [79]. In a detailed analysis based on the core–shell model, the EPR signal at *g* ≈ 2.00 was designated as surface defects [80–82]. As seen from PL and CL measurements (Figs. 2a, 6a), the ZnO NRs in this work emits mainly in the NBE UV region and the defect emission is significantly weak even before the annealing with the addition of citrate in the growth process (Fig. 2b). Therefore, the observed EPR signals at *g* ≈ 1.96 can be attributed to the shallow donors Zn^+^ + Donor (Al, Ga, In) and the disappearance of EPR signal at *g* ≈ 2.00 can be attributed to negligible surface defects. Therefore the *g* ≈ 2.00 signal can be attributed to the paramagnetic signal due to oxygen vacancies and the one at *g* ≈ 1.96 as zinc interstitials as well. [28] Similar observations have been reported on doped ZnO [26, 29, 30, 32, 76, 77].

**Fig. 5:**
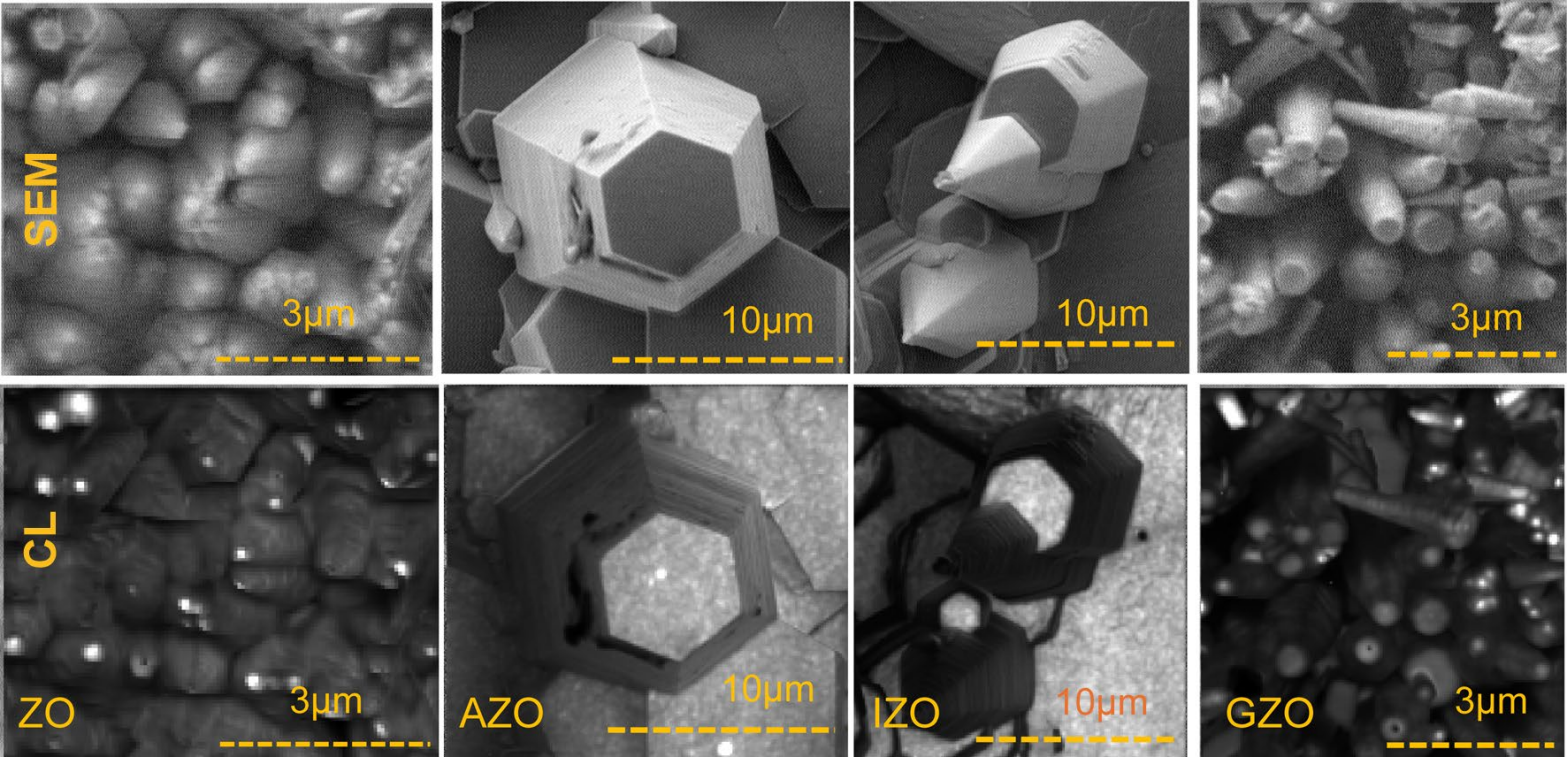
SEM and CL images of the as grown and doped ZnO NRs at 380 nm. All CL plots are using the same color bar to allow for direct comparison

**Fig. 6:**
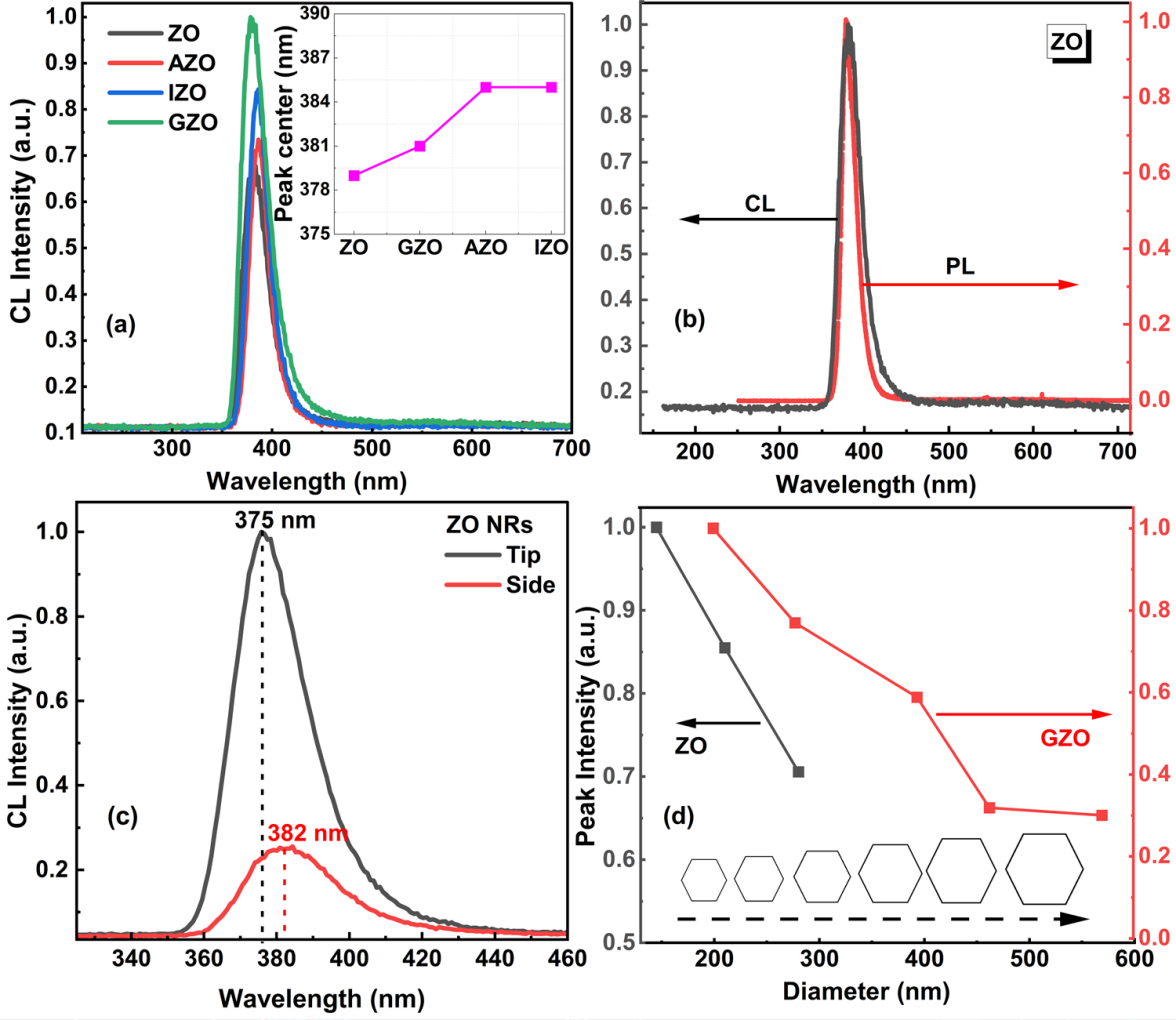
**a** Intensity normalized room temperature CL spectra of a single ZnO NR at core region. Inset shows shift (nm) in peak centers. **b** CL (black) and PL (red) spectra of as grown ZnO NRs. For CL, the spectra were taken on top of the NRs with a 5 kV, 14 pA excitation beam. For PL, a 325 nm He–Cd laser was used as the excitation source. **c** CL emission spectra of a ZO NR at tip and side regions. **d** Variation of CL peak intensities with increasing NR diameter for ZO and GZO NRs. Error bars are not visible for Fig. 6a inset and Fig. 6d

Although the PL measurements give fundamental luminescence characteristics of the crystal structure, they may not represent the optical properties of all regions in a nanostructure. To investigate the possible variation of luminescence intensity in different parts of ZnO NRs and characterize them individually, high spatial resolution (~ 10 nm) cathodoluminescence (CL) spectroscopy with a highly focused electron beam was used. Figure 5 shows typical SEM and CL images of as-grown and doped ZnO NRs. Note that all ZnO NRs have strong NBE UV emission on the tip for highly tapered NRs and on the top surface for non-tapered flat NRs, and the NBE emission is significantly reduced at the lateral surfaces. NRs ending with both tapered tip and flat hexagonal surfaces were selected to study the variation of CL intensity at those sites. The AZO and IZO images indicate the evolution of NRs into microrod-like stem structures, which clearly confirms the fast-lateral growth rate on these structures. This might be due to the affinity of Al and In for OH^−^ and formation of hydroxides as discussed previously. Further studies on the doping effect on ZnO NRs are in progress. Figure 6 displays a detailed representation of NBE UV emission in doped and as-grown NRs for different region and sizes. A similar trend of NBE UV emission in PL measurement results (i.e. increasing UV intensity with doping) was observed in CL measuresssments (Fig. 6a). Substitution of O vacancies (or Zn) with dopant ions increases the crystalline quality and shifts the NBE UV emission peak to longer wavelengths, thus reducing reabsorption of NBE UV emission as well (Fig. 6a inset). PL and CL spectra taken for ZO NRs are depicted in Fig. 6b. Both spectra are almost identical in peak positions and widths, confirming similar UV emission for individual NRs (top surface) and an ensemble of NRs. Moreover, PL and CL spectra have similar peak centers and showed no visible emission indicating NR crystal structures almost free of defects.

The CL spectra taken at the tip and side of the ZO NRs demonstrate that the NBE UV emission is mainly due to the top polar surface (0001) (Fig. 6c). This is in agreement with our previous work which includes ultra-long ZnO NRs with tuned PL emission indicating an intense visible emission at the lateral surfaces and very weak visible emission at the top of the NRs [39]. Similar intense NBE UV emission on nanotips were reported elsewhere [54, 83–85]. There is also a red shift (~ 60 meV) in NBE UV emission at the lateral surface of the NRs. Figure 6d shows CL peak intensity variation with NR diameter in ZO and GZO NRs. The CL emission was maximized at the core of the top hexagonal surface for each NRs, and the NBE UV intensity decreased as the diameter of the NR increased. Increasing the diameter while holding the length constant results in a lower surface-to-volume ratio, which might explain the variation of CL emission with diameter.

**Fig. 7:**
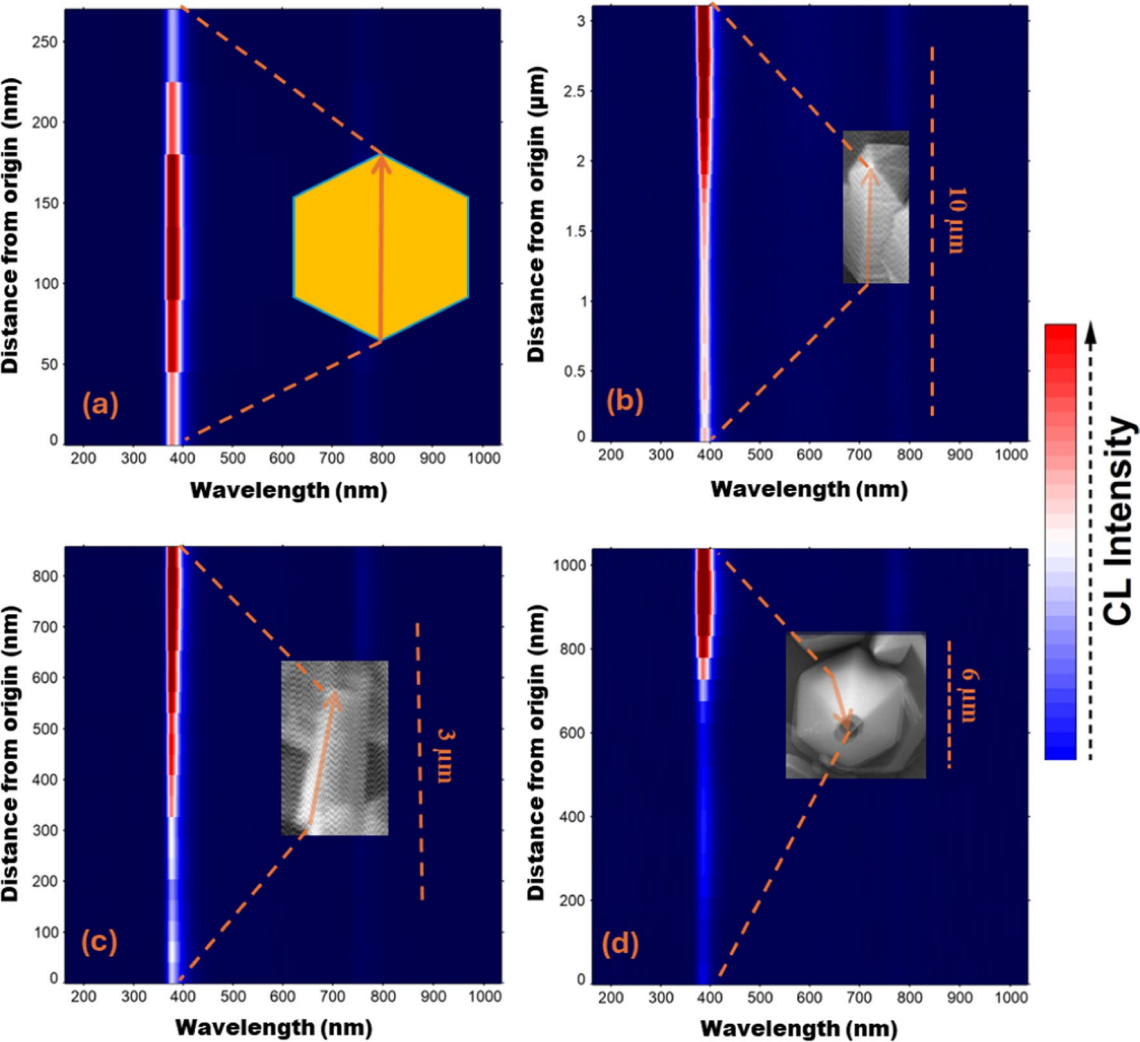
CL intensity map for **a** a ZO NR through the diameter **b** an AZO NR from bottom to the tip **c** a GZO NR from bottom to the top **d** an IZO NR towards the arrow

Spectral CL line scan measurements for individual NR emitters are depicted in Fig. 7. All ZnO NRs exhibit similar luminescence properties with a strong NBE UV emission. However, CL spot and line scan mapping reveals that the spatial distribution of this UV emission is strongly dependent on the NR cross section. The NBE UV emission is highest at the core of the NRs, and it monotonically decreases as it approaches the edge of each NR (Fig. 7a). For the ZnO NRs emitting strong visible emission and weak UV emission, the visible emission intensity was previously found to increase near the edge of the NR apex [39]. This suggests that the spatial variation in NBE UV emission observed here might be caused by relatively higher densities of O vacancies near the edge of the NRs even though the ZnO NR visible emission is negligibly small in this work. Similar variations in spatial distribution of UV and visible emission have been reported elsewhere [86–88]. Spatial variations in UV emission across the lateral surfaces of doped ZnO NRs such as AZO and GZO were also observed (Fig. 7b, c). The NBE UV emission tends to increase close to the tip of the NRs. This UV emission variation for GZO NRs is consistent with the variation observed with changing diameter of the GZO NRs (Fig. 6d) in that the NR cross sections with the lowest observed UV intensity correspond to the largest NR diameters and vice versa (Fig. 7c). These results clearly confirm that NBE emission characteristics vary across the ZnO NR top and lateral surfaces, likely a result of spatially varying O vacancies across the NR.

**Fig. 8:**
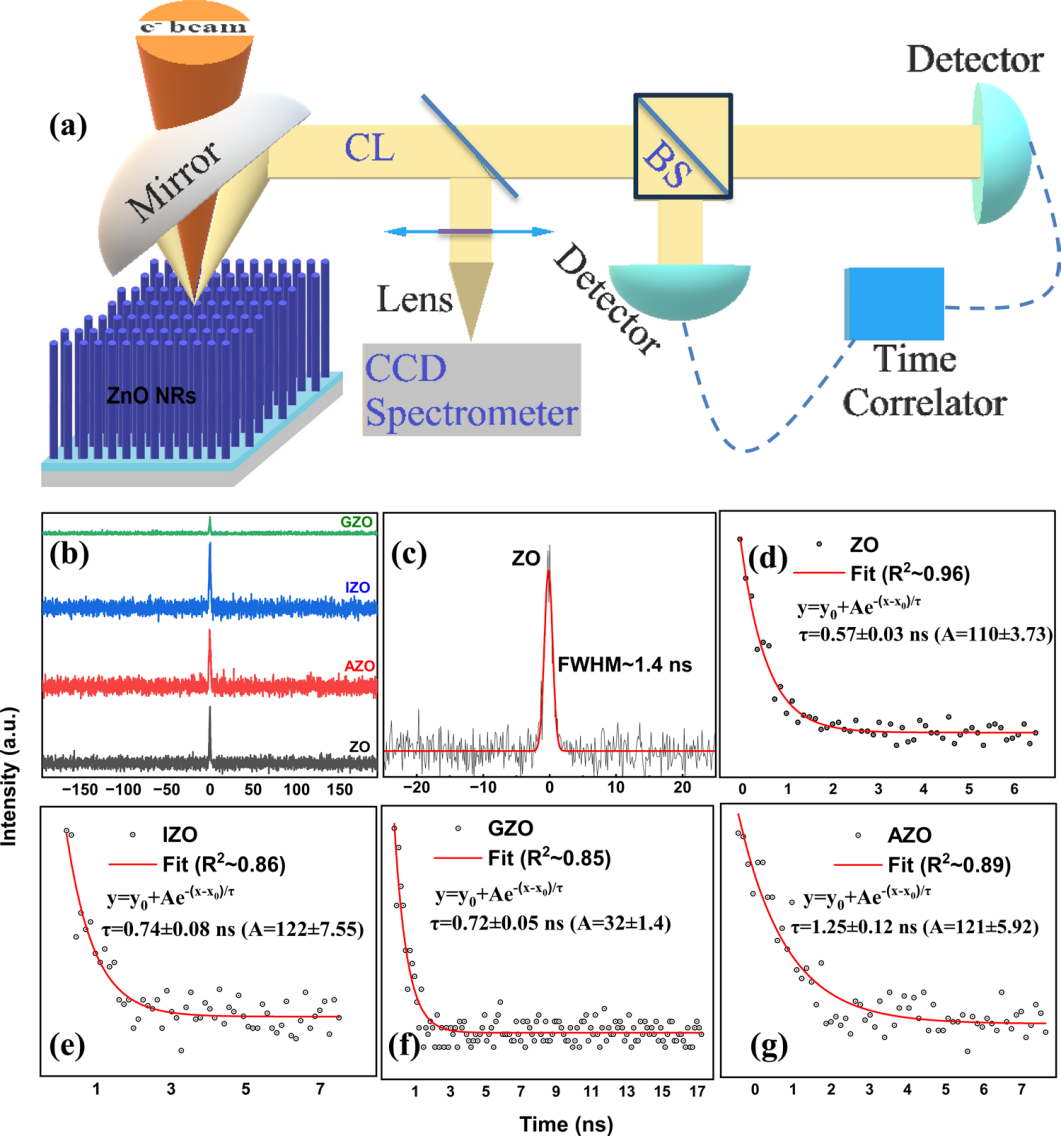
**a** Representation of CL data collection in an SEM instrument: a CCD spectrometer is used to get CL spectrum and photon correlation *g*^2^ (*τ*) was measured using beam splitters and two single photon detectors. Detected photons are time tagged with a time correlator. **b** Raw CL bunching from the as-grown and doped ZnO NR CL **c** Bunching dynamics of ZO NRs **d**–**g** Bunching dynamics at 380 nm for ZO, IZO, GZO, and AZO

In addition to scintillation efficiency, it is very important to develop scintillators with fast decay times for possible use in fast and high-resolution scintillation detectors. To that end, CL photon correlation measurements were performed to evaluate the NBE UV emission dynamics (Fig. 8a). These measurements of the CL second order photon correlation function provide a direct nanoscale probe of the excited state dynamics with no loss in spatial resolution, making this approach appealing compared with time-resolved CL techniques relying on pulsed electron beams [45]. The unnormalized photon correlation measurements are shown for as-grown and doped ZnO in Fig. 8b–g. An IRF with FWHM@ ~ 250 ps was used as a reference (Fig. S8). All samples presented a mono-exponential decay time behavior. The decay time representing the fluorescence lifetime of the electron–hole recombination consists of one component which could be attributed to the free exciton recombination lifetime and also indicates a homogeneous excited state luminescence [10, 89, 90]. The fitted temporal decay curves with exponential decay functions resulted with decay times 0.57, 0.72, 0.74, and 1.25 ns for ZO, GZO, IZO, and AZO, respectively. The relatively slower response of AZO NRs could be related to its evolution to µm scale. Significantly reduced surface-to-volume ratios in AZO NRs might be the reason for the slowest decay time and could indicate that the lifetime of the free exciton is influenced by surface effects [91]. It has been reported that decay time increases as the size increases in ZnO NRs which is consistent with the present results [92–94]. While ZnO was reported to have decay times down to a few tens of ps, they are either thin films or nanorods which have a very short thickness up to a few µm [36, 95]. Since alpha particles have range in the order of tens of µm, the energy deposited in these ZnO samples will be limited which reduces the scintillation efficiency. The decay responses for most of the ZnO NRs in this work are in the sub-nanosecond region and are thick enough to fully stop alpha particles. This indicates that their timing performance could still satisfy the requirement for a fast scintillator and could pave the way for further improvements in time resolution of the radiation detectors.

**Fig. 9:**
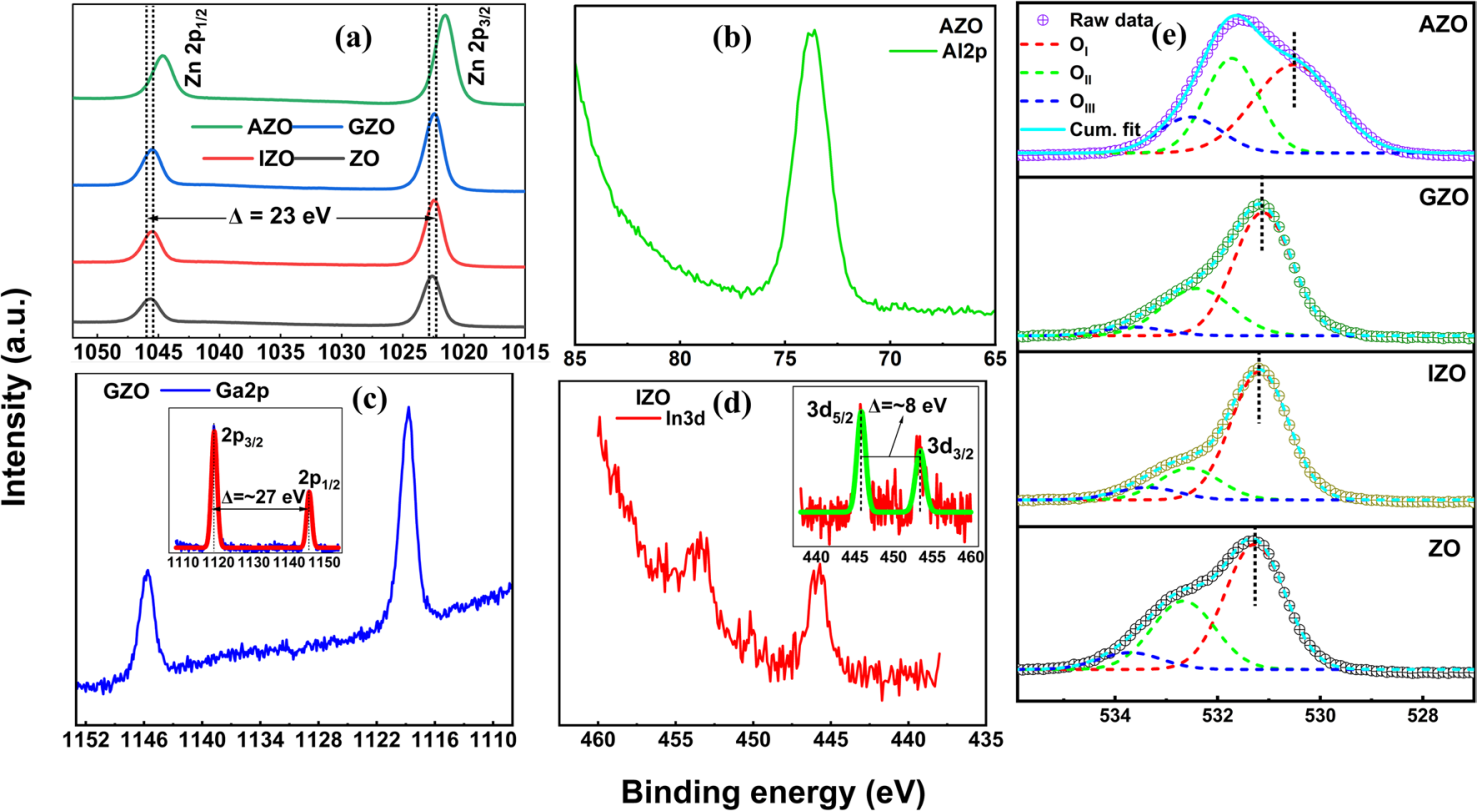
**a** Zn 2p raw XPS spectra of as grown and doped ZnO NRs including the spin-orbital-splitting energy difference between the Zn 2p_3/2_ and Zn 2p_1/2_ **b** Al 2p spectrum of AZO NRs **c** Ga 2p spectrum of GZO NRs **d** In 3d spectrum of IZO NRs **e** deconvoluted XPS spectra of the O 1 s of as grown and doped ZnO NRs

The typical XPS wide survey spectra of unannealed ZnO NRs including Zn, O and C peaks are shown in Fig. S9. Small C peaks observed even after the etching process could originate near surface regions due to the exposure of the NRs to ambient atmosphere or carboxyl groups formed after dissolution of citrate in the solution. Figure 9a shows the comparison of the raw Zn 2p peaks for as-grown and doped ZnO NRs. The observed peaks located at 1045.6, 1045.5, 1045.5, and 1044.7 eV correspond to Zn 2p_1/2_ for ZO, IZO, GZO, and AZO, respectively. Other peaks located at 1022.6, 1022.5, 1022.4, and 1021.5 eV are attributed to Zn 2p_3/2_ for ZO, IZO, GZO, and AZO, respectively. Zn 2p binding energies of doped ZnO NRs were found to be lower than that of as-grown ZnO NRs. While the difference in binding energies for Zn 2p was negligibly small for GZO and IZO when compared to that of ZO, the binding energy for AZO NRs differs significantly (Δ = 0.9 eV for Zn 2p_1/2_, Δ = 1.1 eV for Zn 2p_3/2_) with respect to that of ZO. This asymmetry observed in Zn 2p peaks of AZO NRs could be attributed to the excess amount of Zn in AZO NRs [96]. The binding energy of Zn 2p peaks in AZO was observed to be smaller when compared to that of ZO confirming that the Zn atoms in AZO NRs mainly remain in the valence state of Zn^2+^ [96–98]. Moreover, the electronegativity of Al (*χ* = 1.61) is lower than that of Zn (*χ* = 1.65), hence the valence electron density of Zn in the Zn–O–Al bond in AZO could become higher than that in the Zn–O–Zn bond in ZO. Therefore, the screening effect of the Zn could be more pronounced and might reduce the binding energy of Zn 2p in AZO.

Zn 2p peak analysis showed that the binding energy difference between two peaks is Δ = 23 eV and the ZnO NRs have the + 2 oxidation state. Similar results were reported for ZnO NRs previously [99]. Figure 9b–d shows the Al 2p peak of AZO, Ga 2p peak of GZO, and In 3d peak of IZO, respectively. Those peaks along with ZnO patterned XRD results (Fig. 1b) confirm incorporation of the Al, Ga, and In into the ZnO lattice. The O 1 s peaks of the unannealed ZnO NRs are shown in Fig. 9e. Deconvolution of the O 1 s XPS spectra resulted with three different oxygen species in the surface. O 1 s peaks labeled as O_I_, O_II_, and O_III_ are attributed to lattice oxygen i.e. O^2−^ combined with metal ions, O deficient regions i.e. O vacancies, and adsorbed O i.e. Zn(OH)_2_, respectively [17, 98, 100–102]. The integrated intensity ratio of O_II_ to total O peaks indicated that the doped ZnO NRs might have less O vacancies than the as-grown ZnO NRs except for the AZO NRs for which the surface-to-volume ratio significantly differs from others and might lead to higher oxygen vacancy related surface defects before annealing (Fig. S10a). While the O 1 s peaks display a non-negligible oxygen deficiency peak, they refer to the unannealed samples and the defect related emission became negligible after annealing as confirmed in PL (Fig. 2a) and CL (Fig. 6a) measurements. The FWHM values of fitted O peaks are also shown in Fig. S10b–d.

**Fig. 10:**
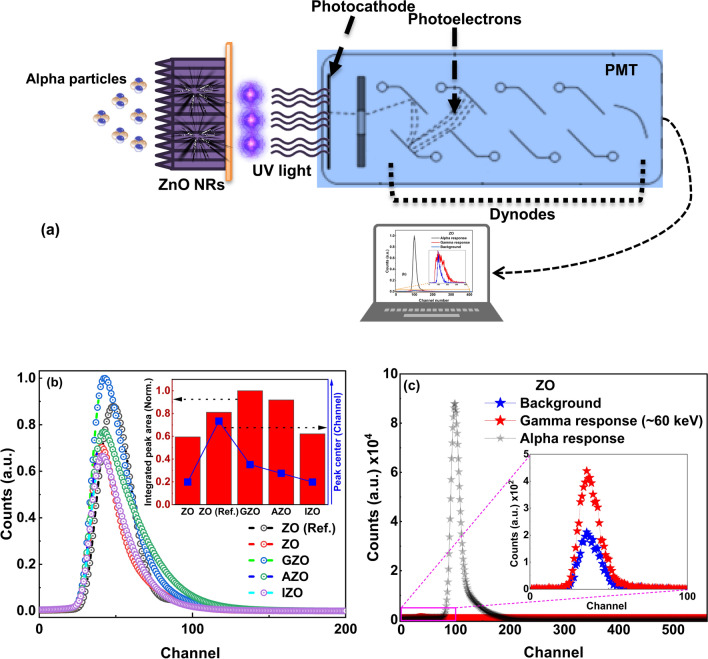
**a** Schematic of the alpha particle response measurements. Pulse height distributions using ^241^Am *α*-particle excitation for **b** as grown and doped ZnO NR scintillators. Inset shows integrated area intensity distribution of ZnO NRs (Error bars are not visible in peak center values). **c** As grown ZnO including gamma response and background

The scintillation properties of ZnO NRs are limited by their structural and optical properties. The spatial resolution of a detector depends on the geometry and structure of the scintillator. Using scintillators consisting of optically isolated structures could help avoid optical crosstalk between adjacent pixels, thus potentially improving the spatial resolution of the imaging detectors [48]. The vertically aligned ZnO NRs could be a good candidate for optically isolated structural design of scintillators as it showed promising scintillation properties such as improved image quality and spatial resolution through simulation and experiments [103–105]. Also, it has been shown in our recent study that the tapered ZnO NRs show excellent anti-reflection properties in the UV region, so it could help light absorption and guiding [11]. The schematic of the alpha particle induced pulse height measurements is shown in Fig. 10a. The alpha particle responses of the ZnO NRs were studied through pulse height measurements using alpha particles of ~ 5.5 MeV from a ^241^Am source. Measurements were conducted in air and the effect of air on alpha particle energy was found to be negligible (Fig. S11b, c). Figure 10b shows pulse height spectra for the ZnO NRs. Measurement parameters including counting time (~ 100 s) were the same for all samples. Almost all doped ZnO NRs demonstrated higher integrated peak areas compared to the as grown ZnO NRs. The integrated pulse height intensity under the photo peak of IZO was found to be slightly higher than that of ZO but much less than those of GZO and AZO (Fig. 10b, inset). The length of IZO NRs is significantly higher than those of other ZnO NRs (Fig. 1d). The range of alpha particles in ZnO material was estimated to be ~ 15 µm using the SRIM software (Fig. S11 [106]. Therefore, the excessive length of IZO NRs (~ 29 µm) may have led to self-absorption of scintillation light before reaching the detector. The typical scintillation measurement result for ZnO NRs is shown in Fig. 10c. The measurement results indicated that ZnO NR scintillators have negligible gamma (~ 60 keV) responses. Also, the scintillation measurements were compared with a ZnO single crystal developed by MOCVD method which was used as a reference in our previous work [4]. The alpha particle pulse height spectra are shifted to slightly higher channels in doped ZnO NRs (except for IZO) and the reference ZnO material (the latter one has the highest channel number), confirming their higher light yield compared with undoped ZnO NRs (Fig. 10b, inset). It has been seen from integrated peak area values that GZO and AZO NRs show better alpha particle responses than even the reference ZnO material, which is a qualitative breakthrough compared to our previous works that either do not include full energy alpha particle induced peaks due to low signal-to-noise ratio (or gamma interference effects) or show less alpha particle response than the ZnO reference material (Fig. 10b, inset) [4, 6].

## Conclusions

In the present work, we have synthesized highly crystalline ZnO NRs doped with Al, Ga, and In using a low-cost hydrothermal method. Structural, optical and scintillation properties of these materials have been investigated thoroughly. Enhanced near band edge (~ 380 nm) luminescence properties have been observed in the doped NRs that the addition of citrate in the growth solution significantly reduced the defect emission even before annealing. The hexagonal wurtzite structure is well maintained for the doped ZnO NRs. Therefore, negligible lattice distortion, thus high crystalline structure has been maintained after doping ZnO with post-transition elements. EPR analysis confirmed a dominant signal at *g*≈ 1.96 for all ZnO NRs. CL results confirmed that NBE emission characteristics vary across the ZnO NR top and lateral surfaces likely as a result of spatially varying O vacancies across the NR. Moreover, sub-nanosecond excitonic dynamics were observed using CL microscopy.

The results in this work demonstrated that the low temperature hydrothermal method is a simple and effective way of fabricating vertically well-aligned ZnO NRs which are well-suited for high scintillation properties. In this method, it is easy to control NR properties by changing precursor and additive concentrations in the solution. Surface defects are highly suppressed through the introduction of citrate as an additive in the growth solution. This additive leads to significantly increased NBE UV emission. Donor doping of ZnO NRs with Al, In, and Ga did not adversely affect the structural and optical properties of the NRs. While all doped ZnO NRs showed better optical and scintillation properties than undoped ZnO NRs, the Ga incorporated ZnO NRs demonstrated highest NBE UV emission and alpha particle response. Together with the superior alpha particle scintillation properties these results indicate that ZnO NRs with n-type doping could be a potential super-fast scintillator to be used in alpha particle scintillator screens.

## Supplementary Information

Supplementary file 1.

## Data Availability

The data supporting this work are accessible upon reasonable request from the corresponding author.
