# Supplementary material for: Synergistic effects of Al, Ga, and In doping on ZnO nanorod arrays grown via citrate-assisted hydrothermal technique for highly efficient and fast scintillator screens
Source: Discov Nano. 2025 Jul 11;20(1):109. doi: 10.1186/s11671-025-04227-5 (PMC12254462; doi:10.1186/s11671-025-04227-5)
Supplement: Supplementary file 1 [file 11671_2025_4227_MOESM1_ESM.docx]

Supplementary information for “**Synergistic Effects of Al, Ga, and In Doping on ZnO Nanorod Arrays Grown via Citrate-Assisted Hydrothermal Technique for Highly Efficient and Fast Scintillator Screens**”


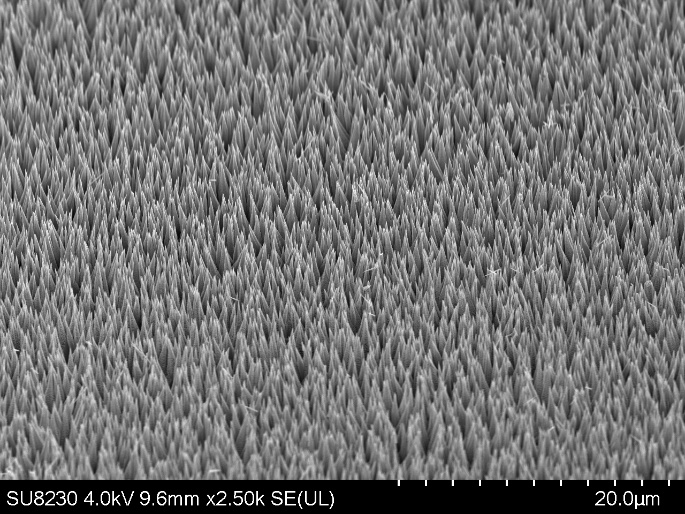

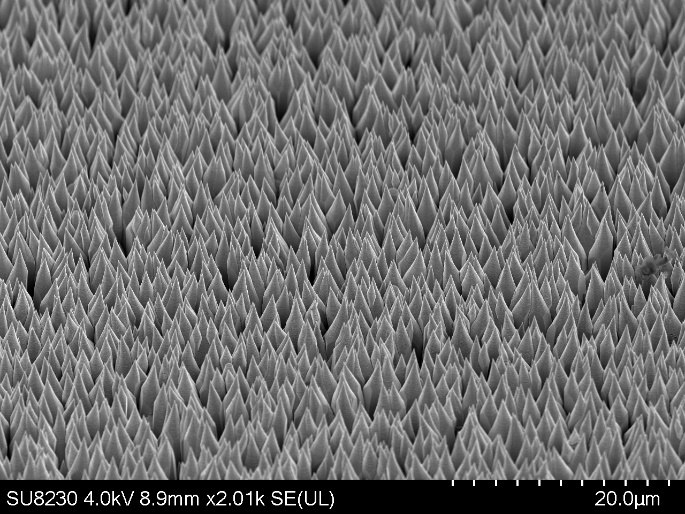


**IZO**

**GZO**


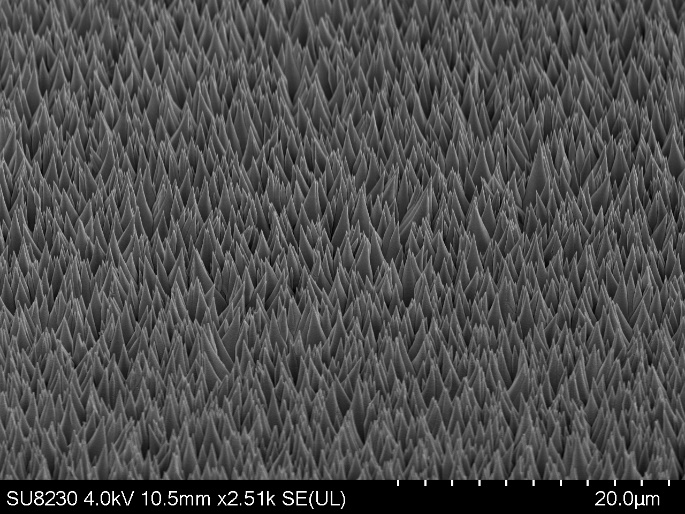

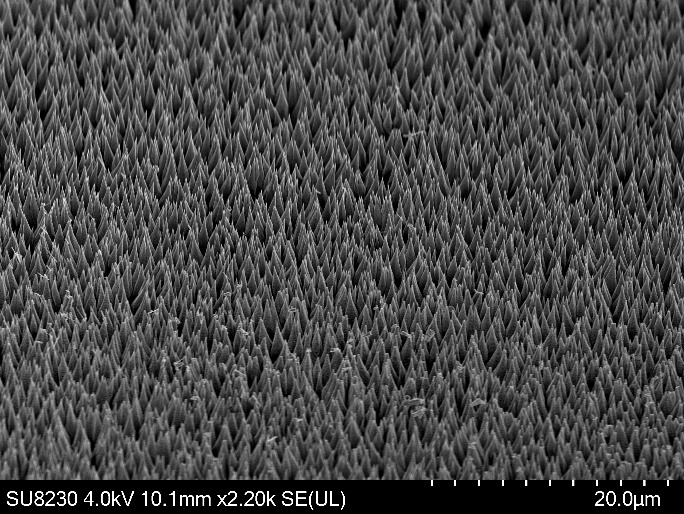


**ZO**

**AZO**

**Figure S1** Tilted top view SEM images of as grown and doped ZnO NRs




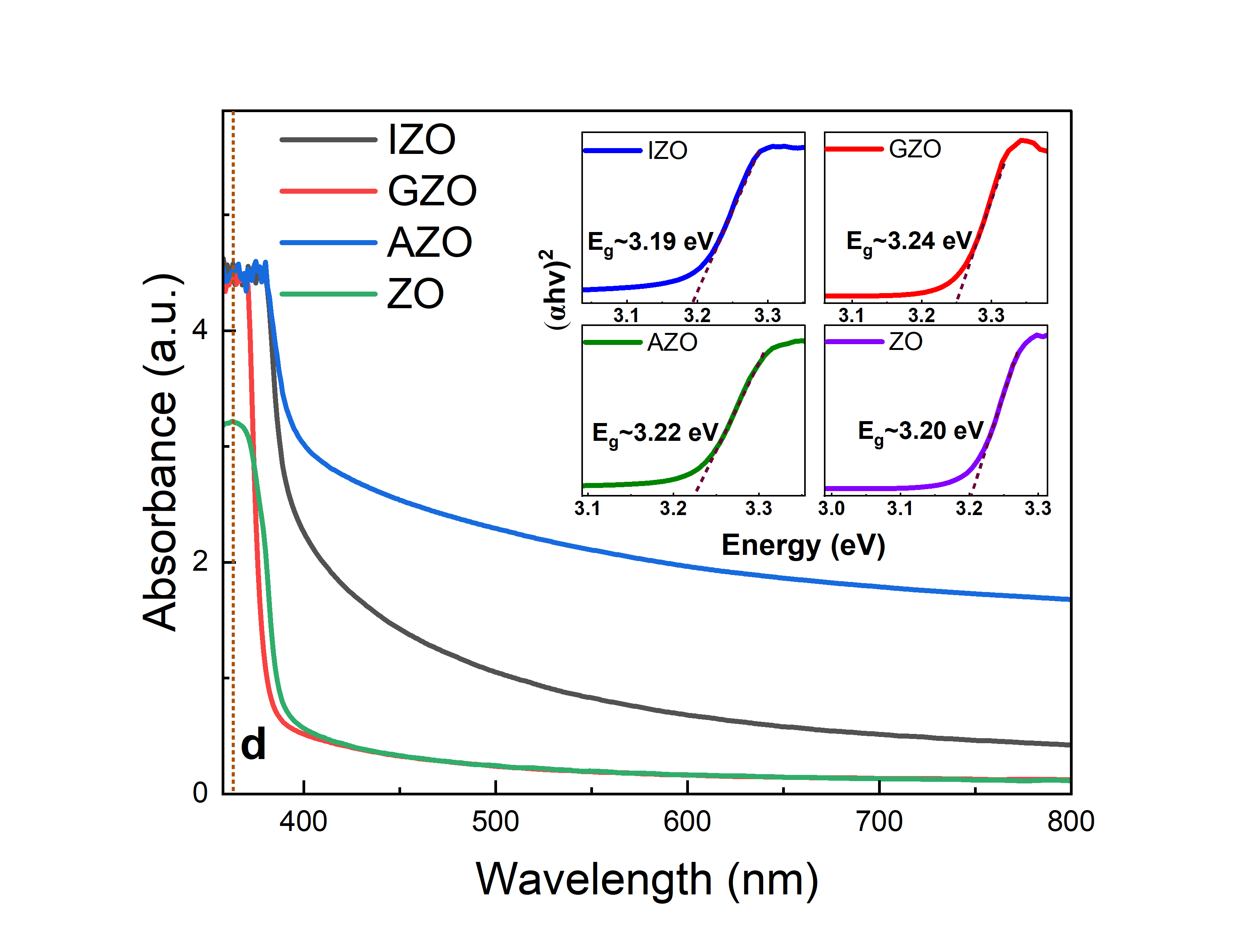


**Figure S2.** a-c) Crystallite size, FWHM, and peak centers derived from XRD data d) UV-vis absorbance spectra and Tauc plots (inset) of as grown and doped ZnO NRs





**Figure S3.** PL spectra of as grown ZnO NRs using different ND filters so as to adjust the PL intensity for convenience. Numbers in D1-4 represent the exponential attenuation factors i.e. D1-0.1, D2-0.01, D3-0.001 etc. The D2 filter was used for optimum PL measurements.





**Figure S4.** PL measurements of as grown ZnO NRs taken at different spots on the sample. Inset shows relative difference in integrated PL intensity of different regions with respect to the region 1.





**Figure S5.** Room temperature steady state PL spectra of ZnO NRs using a Xe lamp

**Figure S6.** Energy levels of native donor (${Zn}_{i}^{\cdot\cdot}, {Zn}_{i}^{\cdot}, V_{O}^{\cdot})$and acceptor ($V_{Zn}^{''})$defects in ZnO NRs based on Kröger Vink notation where *V* and *i* refer to the vacancy and interstitial, respectively. The terms stand for the atomic sites while the superscripts stand for the charges, where a prime and a dot indicate negative and positive charge (charges are proportional to the number of superscripts), respectively.





**Figure S7.** Variation of room temperature NBE PL spectra of as grown ZnO (ZO) NRs with respect to the excitation wavelength a) 255-273 nm b) 279-299 nm c) 301-331 nm d) 333-373 nm.





**Figure S8.** Prompt response (IRF) of the detector used in CL photon correlation measurements (acquired using transition radiation from a gold thin film). Inset shows exponential decay curve.


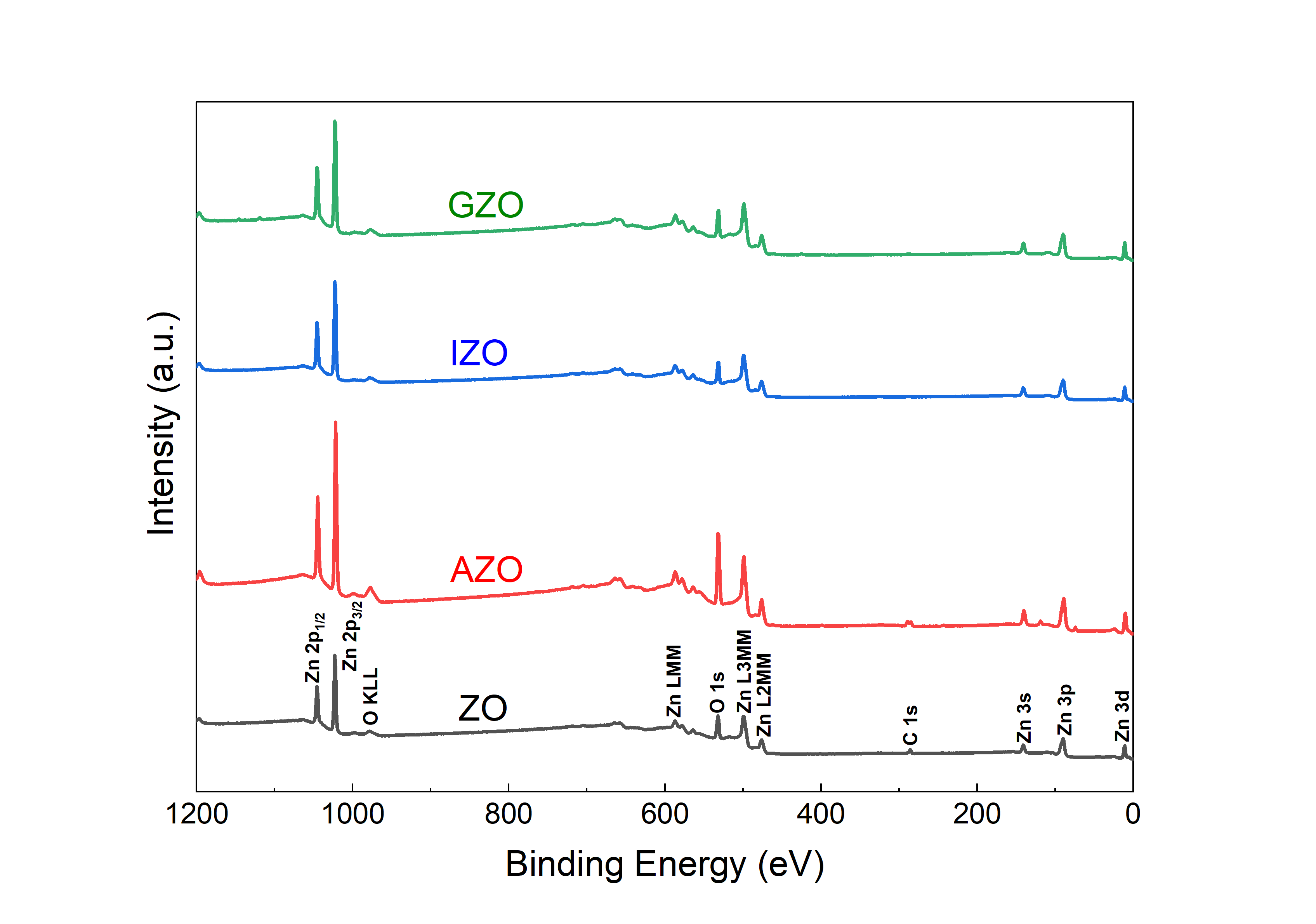


**Figure S9.** XPS wide survey spectra of as grown and doped ZnO NRs





**Figure S10.** a) The intensity ratio of O_II_ to O_Total_ for ZnO NRS b-d) FWHM of O_I_, O_II_, and O_III_ peaks

**
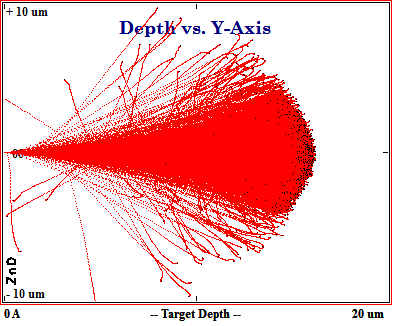

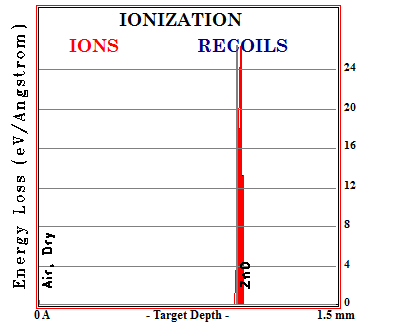

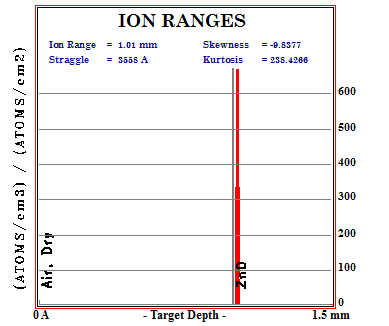
**

**(c)**

**(a)**

**(b)**

**Figure S11.** Simulation of alpha particle transport in ZnO using SRIM software.^[67]^ a) Alpha particle track diagram for ^241^Am source b) Ionization plot confirming that the ionization mostly occurs in ZnO c) Range of alpha particles. ZnO along with 1 mm air was considered in simulations resulting ~15 µm range in ZnO but negligible change in alpha particle energy. The simulation is not an actual representation of NR design but gives an estimate of how alpha particles interact with a solid and full density ZnO material.
